# Supplementary material for: Reservoir computing from collective dynamics of active colloidal oscillators
Source: Commun AI Comput. 2026 Jul 23;1(1):6. doi: 10.1038/s44488-026-00001-3 (PMC13446078; doi:10.1038/s44488-026-00001-3)
Supplement: Supplementary file 1 [file 44488_2026_1_MOESM1_ESM.pdf]

Supplementary Information to Reservoir computing from collective dynamics of active colloidal oscillators

Veit-Lorenz Heuthe[1,2], Lukas Seemann[1], Samuel Tovey[3], and Clemens Bechinger[1,2,†]

[1]Fachbereich Physik, Universität Konstanz, D-78465 Konstanz, Germany
[2]Centre for the Advanced Study of Collective Behaviour, Universität Konstanz, 78464 Konstanz, Germany
[3]Institute for Computational Physics, University of Stuttgart, 70569 Stuttgart, Germany

[†]Corresponding author. Email: clemens.bechinger@uni-konstanz.de

1 Supplementary figures

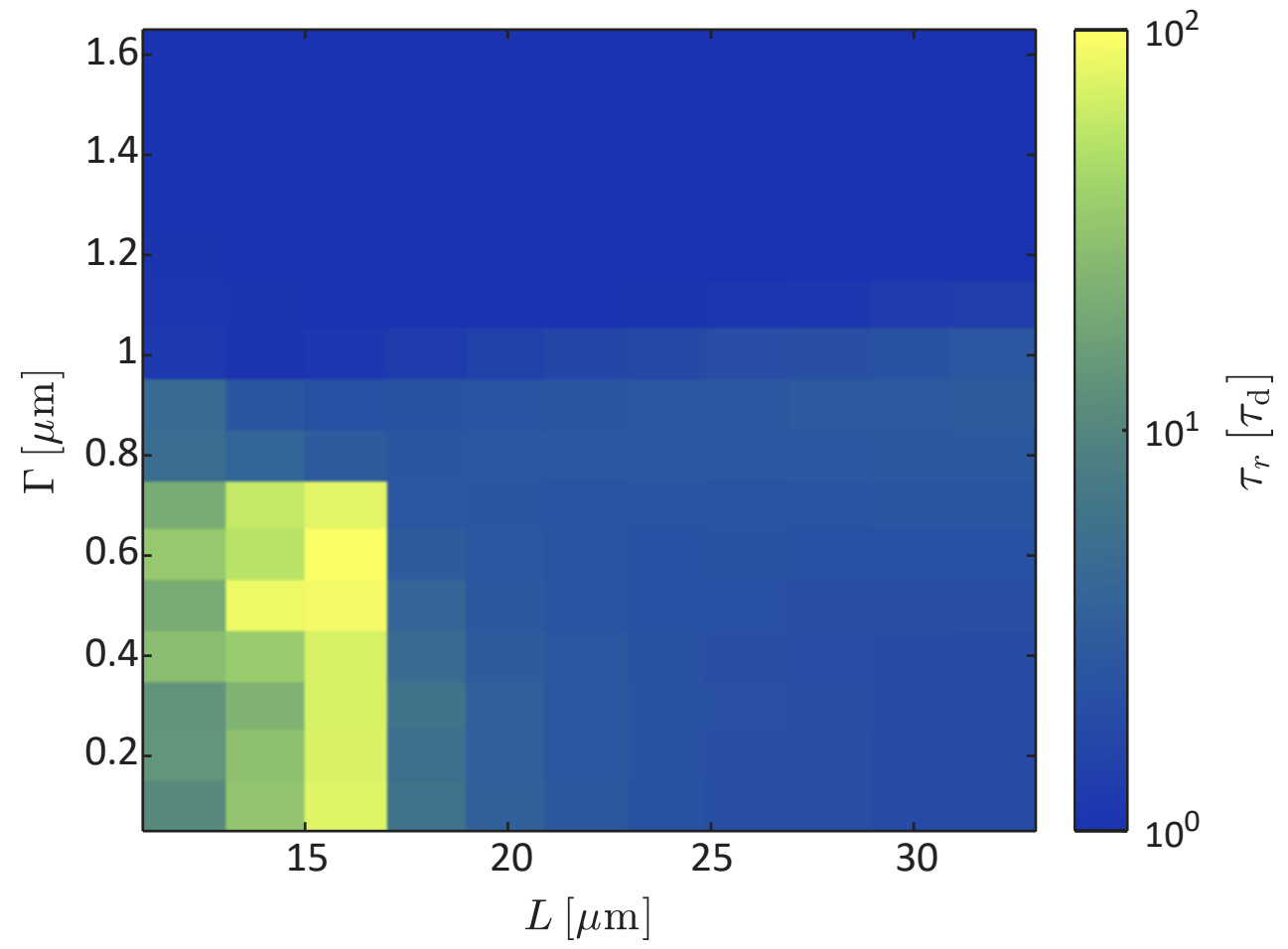

Fig. S1: Relaxation of the reservoir in simulations with arrays of 400 ACOs after excitation by displacing all target positions in the x-direction by $4\,\mu$m for $4\,\tau_\mathrm{d}$.

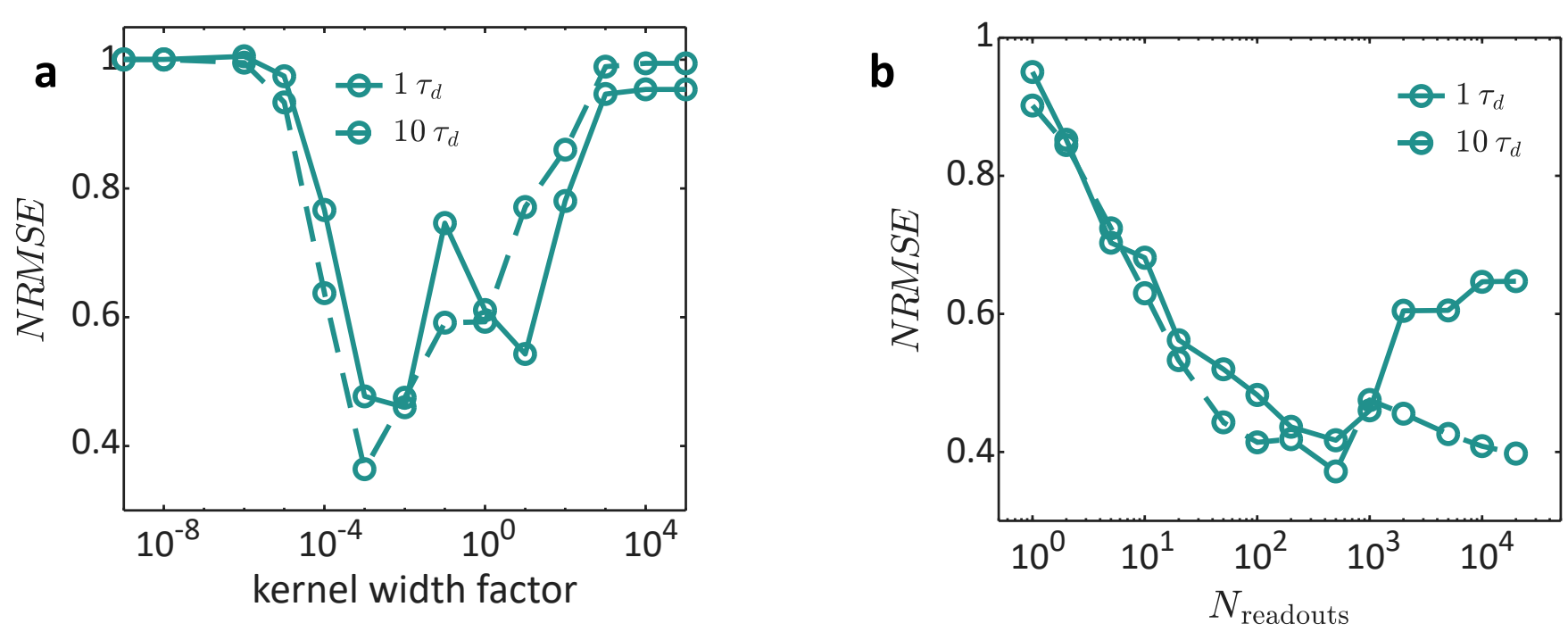

Fig. S2: Optimization of Gaussian kernels. **a** *NRMSE* for Mackey-Glass chaotic time series prediction for different kernel width factors. **b** *NRMSE* for Mackey-Glass chaotic time series prediction for different number of Gaussian kernels, i.e. for different numbers of readouts $N_{readouts}$.

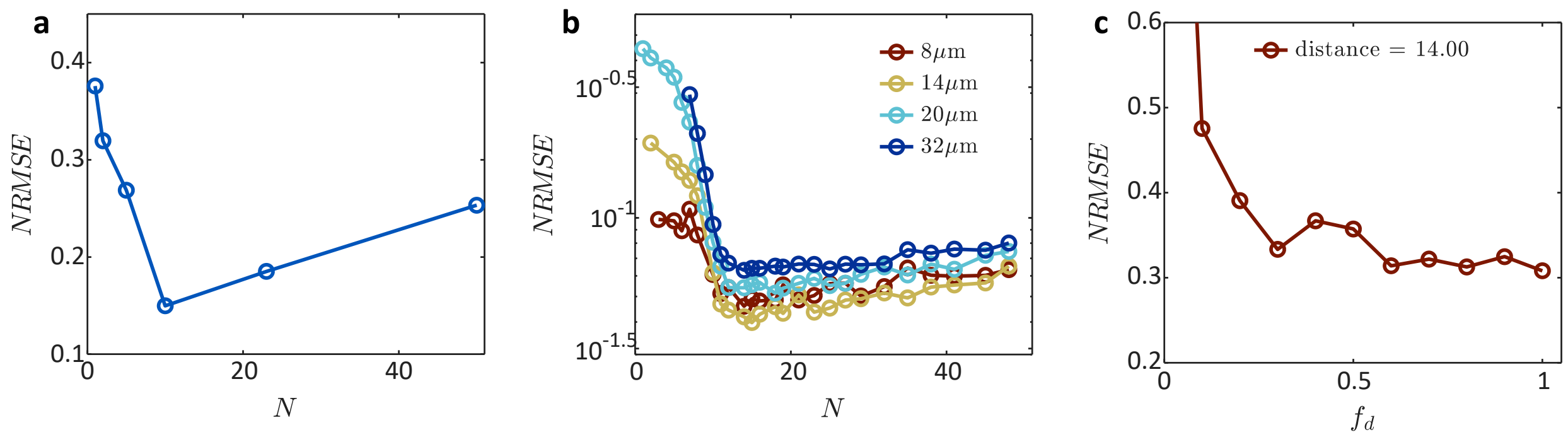

Fig. S3: MG forecasting errors in experiment a Normalized root mean squared error *NRMSE* in experiments as a function of the number N of previous signal states that are fed in to the reservoir. **b** *NRMSE* against the number N of injected states in the reservoir in simulation (extension to Fig. 3f in the main text). **c** *NRMSE* as a function of the fraction f_{driver} of ACOs that receive the input signal in experiments.

2 Memory capacity and nonlinearity

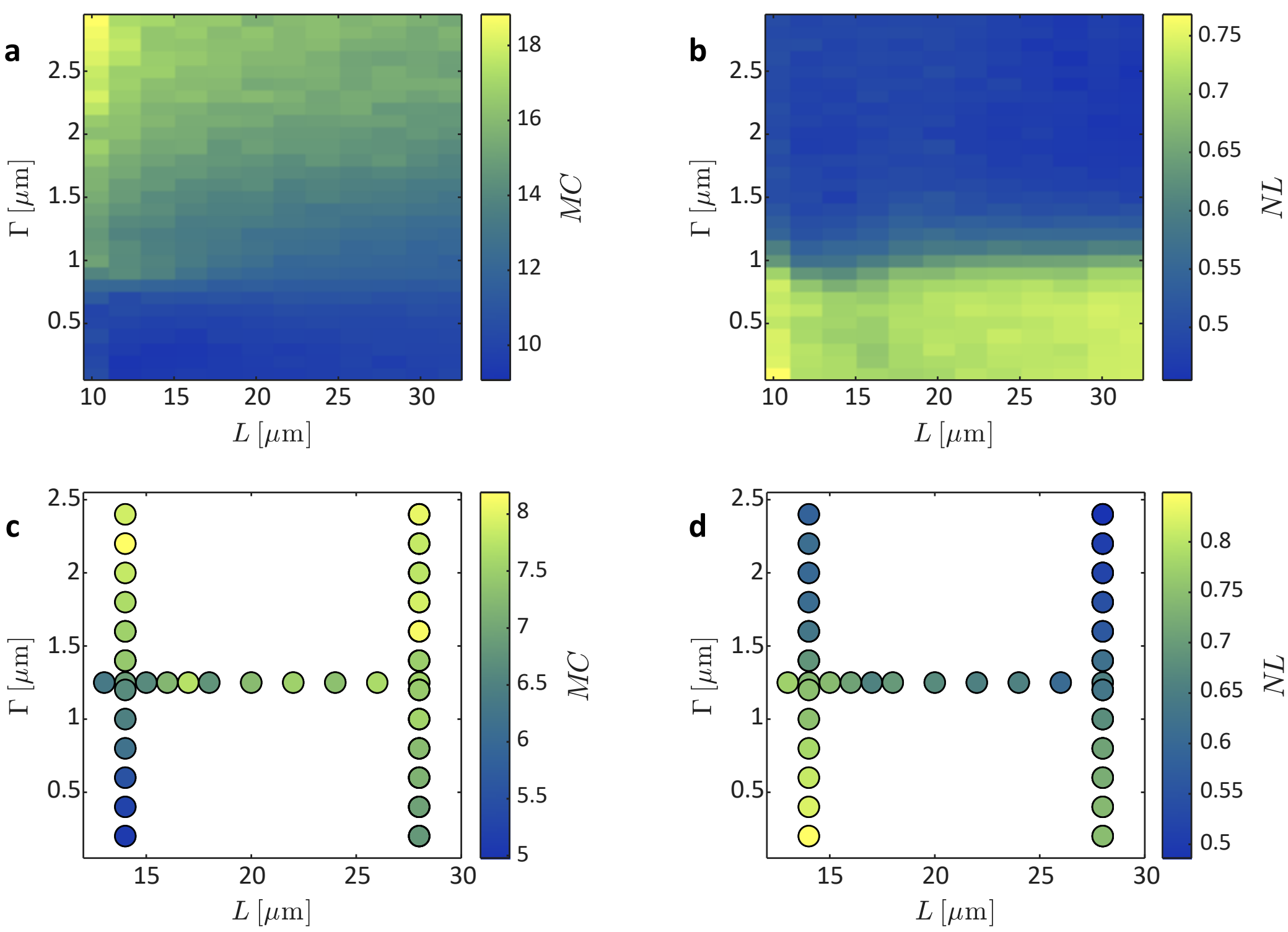

Fig. S4: Memory capacity *MC* **and nonlinearity** *NL* **of the reservoir. a** Memory capacity *MC* as defined in [1] of the ACO arrays as a function of the ACO spacing L and damping parameter Γ determined from simulations. **b** The nonlinearity *NL* as defined in [2] of the ACO arrays as a function of the ACO spacing L and damping parameter Γ determined from simulations. **c** *MC* as a function of L and Γ in experiments. **d** *NL* as a function of L and Γ in experiments.

In the main text, we used the Mackey-Glass chaotic time series prediction accuracy as a measure of the reservoir computing performance of the ACO arrays. However, there also exist task-agnostic measures to asses the computing capabilities of physical systems as reservoir, most importantly *short-term memory capacity MC* and *nonlinearity NL*. To gain a mechanistic understanding of the forecasting performances observed in Fig. 3 in the main text, we have evaluated these quantities following their definitions in [1] and [2] as a function of the ACO spacing L and the damping parameter Γ. All analyses presented in this section were performed with $N = 1$ The memory capacity (Fig. S4a, simulation data) shows a clear dependency on both L and Γ. This dependency indicates that the memory in the ACO arrays is a joint effect of the individual ACO dynamics and

their hydrodynamic interactions, which scale with L. We observe the highest MC for small distances between neighboring ACOs and strong damping. While the system displays long relaxation times at small values of Γ (compare Fig. 2 in the main text), the ACO dynamics are dominated by spontaneous oscillations in this regime, which presumably leads to the reduced MC.

For most of the tested parameter combinations we find $NL \gtrsim 0.5$, showing that the collective dynamics of the ACO arrays possess the required nonlinearity for reservoir computing (see Fig. S4b, simulation data). These values are comparable to other recently presented instances of physical reservoirs [2, 3]. The nonlinearity NL is highest for small Γ and shows a sharp decrease around a damping parameter of $\Gamma = 1\mu$m. This value of Γ matches the oscillation radius of the undamped ACOs. As discussed in the Results section in the main text, Γ is defined as the threshold in the distance to the target, below which an active colloid is propelled with a reduced laser intensity. We interpret the step-like decrease of NL around this value as a result of the threshold distance Γ exceeding the natural oscillation radius.

The experimental results for MC and NL (see Fig. S4c-d) follow similar trends as the simulation results. The memory capacity in the experiment is significantly lower than in the simulations, which we attribute to stronger noise and imperfections of the active colloids in experiment, such as temporary unresponsiveness or phoretic clustering. We conclude that noise and imperfections are the main reasons for the lower prediction performance in the experiment compared to the simulations, which aligns with the findings in [4]. Interestingly, the nonlinearity is larger in experiment than in simulations. While in simulations L does not have a large influence on the nonlinearity, NL increases with smaller distance between ACOs in the experiment. In combination, these findings indicate that the hydrodynamic interactions between ACOs contribute to the nonlinearity of the system, and these contributions are under-estimated in simulations due to an idealized interaction model.

Noticeably, MC and NL seem to show opposing dependencies on L and Γ. This so-called memory nonlinearity tradeoff is a well known characteristic of reservoirs and has for example been discussed in [5] and [6]. Neither MC nor NL shows a maximum for the parameter combination at which we obtained maximal forecasting accuracy ($L = 14\ \mu$m, $\Gamma = 1.5\ \mu$m, see Fig. 3d in the main text). Instead, the reservoir performance is maximal for parameters that balance memory capacity and nonlinearity. When either MC or NL is very low, we observe low reservoir performances, highlighting that both properties are necessary for computing.

3 Chaotic Lorenz attractor trajectory forecasting

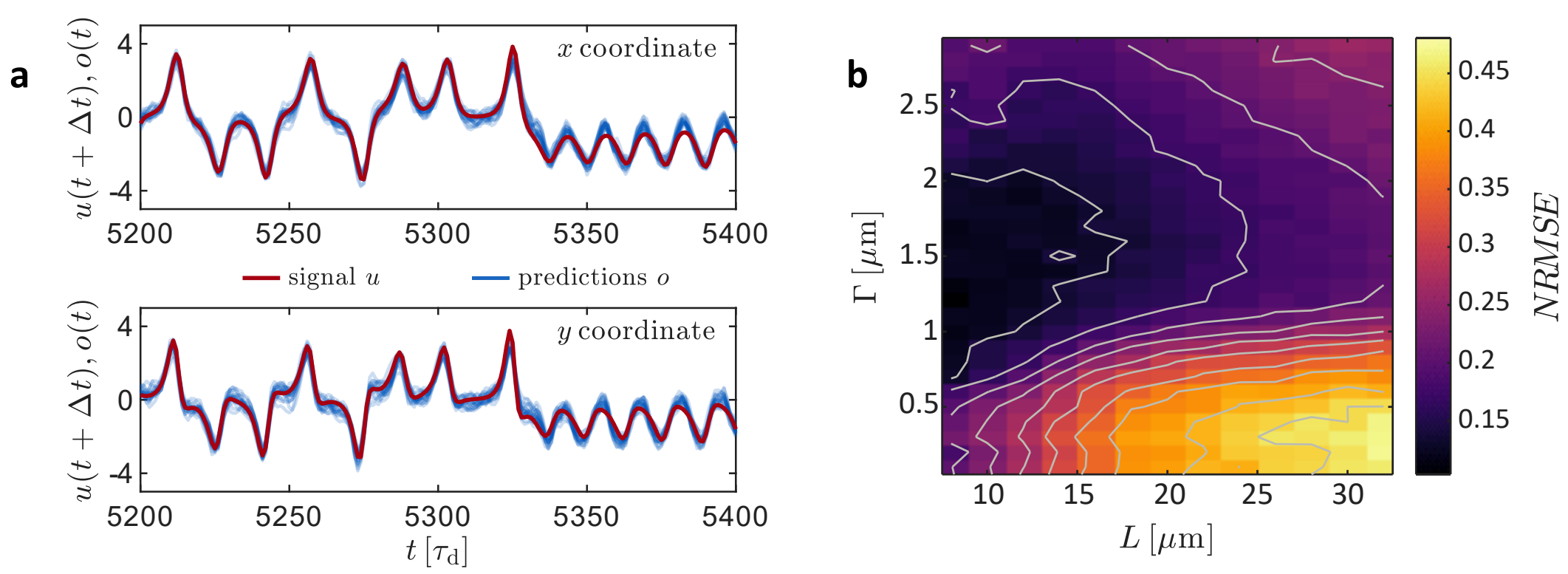

Fig. S5: Forecasting of the chaotic Lorenz63 attractor a Signal (red) and predictions (blue) for x- and y-coordinate (top and bottom, respectively) of the chaotic Lorenz63 attractor in experiments. **b** $NRMSE$ for forecasting the x-coordinate of the Lorenz63 system with $\Delta t = 2\tau_d$ in simulations as a function of the distance L between ACOs and damping parameter Γ.

In addition to the chaotic Mackey-Glass (MG) time series forecasting benchmark presented in the main text (Fig. 3), we have tested the ACO reservoir in predicting the Lorenz63 differential equation system, which is another well established reservoir computing benchmark (for parameters see Methods). Figure S5a shows predictions of the x- and y coordinate from experiments, where the target position of each ACO was driven with the two-dimensional x-y signal of the Lorenz63 attractor. Figure S5b shows the $NRMSE$ for predicting the x-coordinate of the Lorenz63 system in simulations as a function of the separation L between the ACOs in the array and the damping parameter Γ for $\Delta t = 2\tau_d$. Qualitatively, the prediction performance shows the same dependence on these two parameters as for the MG forecasting task (compare to Fig. 3d in the main text), which shows that the observed effects are independent of the task but depend on the properties of the reservoir.

4 Supplementary Movies

- Supplementary Movie 1: **Active colloidal oscillators (ACOs) for reservoir computing.** Microscope video of an active colloid oscillating around its target position (shown in red) and an illustration of how the input signal is coupled to the oscillators.

- Supplementary Movie 2: **Chaotic time series forecasting with ACO arrays.** Microscope video of an ACO array (target positions animated in red), which is used as a reservoir for chaotic Mackey-Glass time series forecasting. The input to the reservoir and the readout using Gaussian kernels is illustrated together with the input signal, prediction and prediction errors.

- Supplementary Movie 3: **Anomaly detection with ACO arrays.** Microscope video of an ACO array (target positions animated in red), which is used to detect anomalies in a chaotic Mackay-Glass time series.

References

1. Jaeger, H. Short term memory in echo state networks, GMD Report 152 (2002).
2. Lee, O. *et al.* Task-adaptive physical reservoir computing. *Nature Materials* **23,** 79–87 (Jan. 2024).
3. Das, S. *et al.* *Reservoir computing in a lithium-based magneto-ionic device* arXiv:2511.08346 [physics]. Nov. 2025.
4. Wang, X. & Cichos, F. Harnessing synthetic active particles for physical reservoir computing. *Nature Communications* **15,** 774 (Jan. 2024).
5. Inubushi, M. & Yoshimura, K. Reservoir Computing Beyond Memory-Nonlinearity Trade-off. *Scientific Reports* **7,** 10199 (Aug. 2017).
6. Dambre, J., Verstraeten, D., Schrauwen, B. & Massar, S. Information Processing Capacity of Dynamical Systems. *Scientific Reports* **2,** 514 (July 2012).
